# The gut microbiota and metabolite profiles are altered in patients with spinal cord injury

**DOI:** 10.1186/s13041-023-01014-0

**Published:** 2023-02-20

**Authors:** Ganggang Kong, Wenwu Zhang, Siyun Zhang, Jiewen Chen, kejun He, Changming Zhang, Xi Yuan, Baoshu Xie

**Affiliations:** 1 Guangdong Provincial Key Laboratory of Orthopedics and Traumatology, Department of Spinal Surgery, The First Affiliated Hospital of Sun Yat-Sen University, Guangzhou, China; 2 Department of Rehabilitation Medicine, The First Affiliated Hospital of Sun Yat-Sen University, Guangzhou, China; 3 Department of Neurosurgery, The First Affiliated Hospital of Sun Yat-Sen University, No. 58, Zhong Shan Er Lu, Guangzhou, 510080 Guangdong China; 4 Department of Anesthesiology, Bazhong Central Hospital, Bazhong, China

**Keywords:** Spinal cord injury, Gut microbiota, 16S rRNA gene sequencing, Untargeted metabolomics

## Abstract

**Background:**

Metabolites secreted by the gut microbiota may play an essential role in microbiota–gut–central nervous system crosstalk. In this study, we explored the changes occurring in the gut microbiota and their metabolites in patients with spinal cord injury (SCI) and analyzed the correlations among them.

**Methods:**

The structure and composition of the gut microbiota derived from fecal samples collected from patients with SCI (*n* = 11) and matched control individuals (*n* = 10) were evaluated using 16S rRNA gene sequencing. Additionally, an untargeted metabolomics approach was used to compare the serum metabolite profiles of both groups. Meanwhile, the association among serum metabolites, the gut microbiota, and clinical parameters (including injury duration and neurological grade) was also analyzed. Finally, metabolites with the potential for use in the treatment of SCI were identified based on the differential metabolite abundance analysis.

**Results:**

The composition of the gut microbiota was different between patients with SCI and healthy controls. At the genus level, compared with the control group, the abundance of *UBA1819*, *Anaerostignum*, *Eggerthella*, and *Enterococcus* was significantly increased in the SCI group, whereas that of *Faecalibacterium*, *Blautia*, *Escherichia*–*Shigella*, *Agathobacter*, *Collinsella*, *Dorea*, *Ruminococcus*, *Fusicatenibacter*, and *Eubacterium* was decreased. Forty-one named metabolites displayed significant differential abundance between SCI patients and healthy controls, including 18 that were upregulated and 23 that were downregulated. Correlation analysis further indicated that the variation in gut microbiota abundance was associated with changes in serum metabolite levels, suggesting that gut dysbiosis is an important cause of metabolic disorders in SCI. Finally, gut dysbiosis and serum metabolite dysregulation was found to be associated with injury duration and severity of motor dysfunction after SCI.

**Conclusions:**

We present a comprehensive landscape of the gut microbiota and metabolite profiles in patients with SCI and provide evidence that their interaction plays a role in the pathogenesis of SCI. Furthermore, our findings suggested that uridine, hypoxanthine, PC(18:2/0:0), and kojic acid may be important therapeutic targets for the treatment of this condition.

## Introduction

Spinal cord injury (SCI), usually resulting from severe trauma such as falls and traffic accidents, is one of the most severe forms of injury to the central nervous system (CNS). SCI is associated with a high rate of disability and serious complications [1, 2]. Patients with SCI typically present with severe neurogenic intestinal dysfunction due to intestinal denervation [3]. In addition to changes in bowel habits, such as the occurrence of constipation and diarrhea, the gut microbiota of patients with SCI is also significantly disturbed, which has a marked impact on the quality of life of affected individuals [4]. The relationship between SCI and intestinal dysfunction has been extensively studied with the aim of developing novel and effective management methods for intestinal impairment. The CNS affects intestinal function via several mechanisms, including the regulation of intestinal motility, intestinal transport time, intestinal permeability, and hormone secretion [5]. Consequently, abnormal gastrointestinal function in patients with SCI can lead to changes in intestinal permeability and, consequently, the migration of intestinal bacteria to the bloodstream and dysbiosis [6]. Although the gut microbiome is thought to be confined to the intestinal lumen, it has been shown that it can also modulate the function of distant organs [7]. These observations highlight the importance of investigating the relationship between SCI and gut microbiota composition.

The gut microbiota is characterized as a collection of microorganisms that colonize the digestive tract. It is an indispensable “microbial organ” in the human body and plays a vital role in the health of the host, including in the CNS [8–11]. For decades, studies investigating the possible impact of microbes and viruses in SCI have been largely constrained by technical limitations; however, with the development of gut microbiota sequencing technology, the correlation between the complex gut microbiome and the CNS has been gradually revealed [12]. The gut microbiome and its metabolites regulate the normal development of the CNS, such as blood–brain barrier formation, myelination, neurogenesis, and microglia maturation [13, 14]. The gut microbiome produces neuroactive metabolites that can cross the intestinal barrier and enter the systemic circulation, where they can influence neural activity and promote neuroinflammation [15, 16]. A balanced microbiome is critical for its symbiotic relationship with the host. Dysbiosis occurs when the composition of the gut microbiome changes, particularly when there are fewer non-pathogenic bacteria or more pathogenic, proinflammatory bacteria. Evidence garnered over recent years has indicated that gut dysbiosis is related to secondary injury and the clinical symptoms of SCI [6, 17]. Studies in mice have shown that the gut microbiome becomes dysregulated after SCI, which exacerbates nerve injury and spinal cord pathology [18, 19]. Dysregulation of the gut microbiome activates the TLR4/Myd88 signaling pathway, which has been reported to aggravate SCI [19].

The exact mechanisms by which gut dysbiosis regulates SCI and the biological mediators of these effects remain largely unknown. Studies have shown that short-chain fatty acids (SCFAs), the primary metabolites produced by bacterial fermentation of dietary fiber, may regulate brain function through immune, endocrine, vagal, and other humoral pathways [20]. In addition, it has been reported that the metabolite 4-ethylphenyl sulfate influences the behavior of mice by affecting oligodendrocyte function and myelin patterning [21]. These findings suggest that gut microbiome-derived metabolites are important mediators of microbiota–gut–brain crosstalk. However, relatively few studies have investigated the changes in overall metabolite abundance in patients with SCI.

In this study, we conducted an omics analysis of gut microbiota structure in patients with SCI as well as a quantitative analysis of metabolites in serum samples. We further examined the relationship between the gut microbiota and changes in serum metabolites.

## Materials and methods

### Study design and sample collection

Eleven patients with SCI and 10 healthy individuals were recruited for this study. The inclusion criteria for the SCI group were (1) cervical or thoracic SCI confirmed via medical history and imaging examination; (2) aged 14 years or more; and (3) American Spinal Cord Injury Association (ASIA) neurological function scale ranging from A to D. The exclusion criteria were the presence of cauda equina injury, infectious disease, severe digestive disease, tumor, diabetes, immune metabolic disease, craniocerebral injury, and mental disorder; and patients receiving antibiotics or probiotics one month before the study. Healthy individuals (age > 14 years) were recruited based on the same exclusion criteria.

Data relating to gender, age, ASIA grade, injury duration, and site of enrolment were collected. Neurological function grade was based on the standardized ASIA Impairment Scale, as previously described [22].

### Sample collection and preparation

Approximately 10 g of fresh stool samples were collected from SCI patients and healthy individuals using sterile plastic spoons and placed in test tubes. Fresh stool samples were frozen at − 80 °C for 16S rRNA gene sequencing within 2 h of collection. Venous blood samples (2 mL) were obtained from patients with SCI and healthy individuals and centrifuged at 3,000 × *g* for 10 min for serum extraction. The serum samples were stored at − 80 °C for UPLC–Q–TOF/MS analysis. The data were analyzed on the free online Majorbio cloud platform (www.majorbio.com).

### 16S rRNA amplicon sequencing

Total genomic DNA was extracted from each sample and purified using the cetyltrimethyl ammonium bromide (CTAB) method [23]. DNA purity and concentration were determined by agarose gel electrophoresis. The V3–V4 region of the 16S rRNA gene was PCR amplified using specific primers. PCR was performed in a 30-μL reaction volume, which included 15 μL of Phusion High-Fidelity PCR Master Mix (New England Biolabs, Ipswich, MA, USA), 0.2 μM of each of the forward and reverse primers, and 10 ng of template DNA. The PCR products were analyzed using 2% agarose gel electrophoresis, purified with an AxyPrepDNA Gel Extraction Kit (Axygen Bioscience, Union City, NJ, USA), and subjected to paired-end sequencing on an Illumina MiSeq/HiSeq 2500 platform.

Reads were clustered into operational taxonomic units (OTUs) at a 97% sequence similarity threshold based on Ribosomal Database Project (RDP) classification. Beta diversity analysis, including principal component analysis (PCA), principal coordinate analysis (PCoA), and partial least squares discriminant analysis (PLS-DA), was performed with the Quantitative Insights into Microbial Ecology (QIIME) software package. Differences between groups were analyzed using *T*-tests, linear discriminant analysis (LDA) effect size (LEfSe), and analysis of similarity (ANOSIM).

### Serum metabolomics

Serum samples (100 µL) were centrifuged at 14,000×*g* for 20 min at 4 ℃ with an equal volume of pre-cooled acetonitrile/methanol (1:1, *v*/*v*) and the supernatant was collected. For LC–MS analysis, the samples were separated using ultra-high-performance liquid chromatography (UHPLC, 1290 Infinity LC, Agilent Technologies, Santa Clara, CA, USA). Electrospray ionization (ESI) was used for detection in both positive and negative ion modes. Mass spectrometric analysis and metabolite identification were performed using an Agilent 6550 iFunnel Q–TOF spectrometer (Agilent Technologies, Santa Clara, CA, USA) and a Triple TOF 6600 mass spectrometer (SCIEX, Framingham, MA, USA), respectively.

The raw data were converted to the mzXML format using ProteoWizard (http://proteowizard.sourceforge.net/) and imported into XCMS software for further analysis, including retention time correction, peak alignment, and picking. Following Pareto-scaling preprocessing, the data were subjected to multivariate (PCA, PLS-DA, and orthogonal PLS-DA [OPLS-DA]) and univariate (fold change [FC] and *T*-tests) analysis. Mean metabolite concentrations in each group were used to calculate FC values. Differentially expressed metabolites were identified using variable importance in projection (VIP) scores > 1 and *p* < 0.05 as criteria.

### Correlation analysis

Correlations among the abundance of gut microbiota at the genus level, the levels of serum metabolites, injury duration, and neurological grading were visualized as a heatmap constructed using SCIPY (Python; Version 1.0.0).

### Statistical analysis

Data were analyzed using SPSS, version 22 (IBM, Armonk, NY, USA). Continuous variables were expressed as means ± standard deviation and independent samples *t*-tests were employed for comparisons between groups. Categorical variables were expressed as rates and the chi-square test was used for comparisons between groups. *p*-values < 0.05 were considered significant.

## Results

### Baseline data for the two groups

The participants, all from Guangdong Province, had similar dietary habits and were given standard dietary guidance for three days before the study. There were no significant differences in age and gender between the SCI group and the control group, which minimized the influence of confounding factors on the study results. Detailed data for the SCI patients are shown in Table 1.

**Table 1:** Comparison of baseline data between patients with SCI and healthy controls

	Control (*n* = 10)	SCI (*n* = 11)	Statistics	*p*-value
Year	40.70 ± 14.41	49.00 ± 20.51	*t* = − 1.062	0.301
Gender (male/female)	6/4	6/5	–	1.000
Injury duration (months)	0	22.81 ± 1.15	*t* = 0.836	0.405
Injury site				
Cervical cord	NA	5		
Thoracic cord	NA	6		
ASIA grade				
A	0	4		
B	0	0		
C	0	3		
D	0	4		
E	10	0		

### The gut microbiota profiles of the two groups

**Fig. 1:**
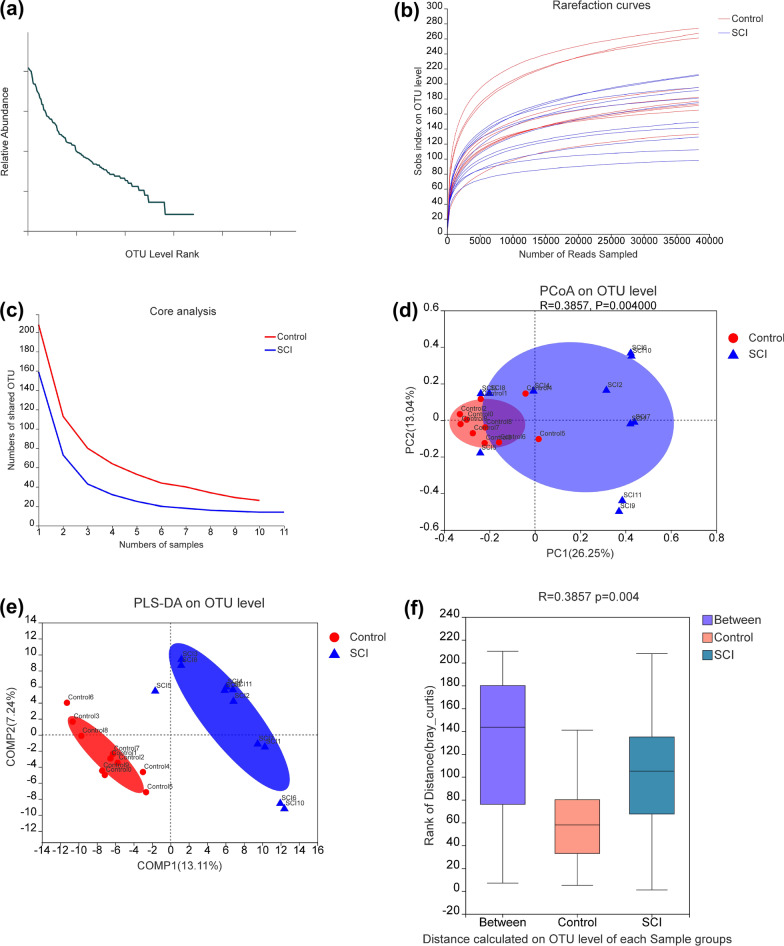
Detection of fecal sample quality and differences in gut microbiota composition between groups. **a** Rank–abundance curves for fecal samples from the control and spinal cord injury (SCI) groups. The abscissa represents the rank of the number of operational taxonomic units (OTUs) and the ordinate represents the relative percentage of OTU number. **b** Sobs index of rarefaction curves at the OTU level between the two groups of samples detected using a 97% similarity threshold. **c** Core curves. The horizontal axis represents the number of observed samples and the vertical axis represents the number of all core species at the OTU level. **d** Principal coordinate analysis (PCoA) score plots. **e** Partial least squares discriminant analysis (PLS-DA) score plots. **f** Weighted UniFrac distances

To investigate whether the gut microbiota profile was changed in patients with SCI, 16S rRNA gene sequencing was performed on fecal samples from both the SCI and Control groups. A total of 1,900,745 sequences were obtained. The OTU similarity level for index assessment was 97%. The richness and evenness of the gut microbiome of the two groups were analyzed using rank–abundance curves (Fig. 1a). The rarefaction curve had obvious asymptotes, the OUT coverage was 98.98% (Fig. 1b), and the core species curve had leveled off (Fig. 1c). These results indicated that the community was adequately sampled. Beta diversity analysis (PCoA and PLS-DA) results showed a significant separation of the gut microbiota between the SCI and Control groups (Fig. 1d, e). ANOSIM analysis demonstrated that the gut microbiota composition of the two groups was statistically different, suggesting that SCI induced gut dysbiosis (Fig. 1f).

**Fig. 2:**
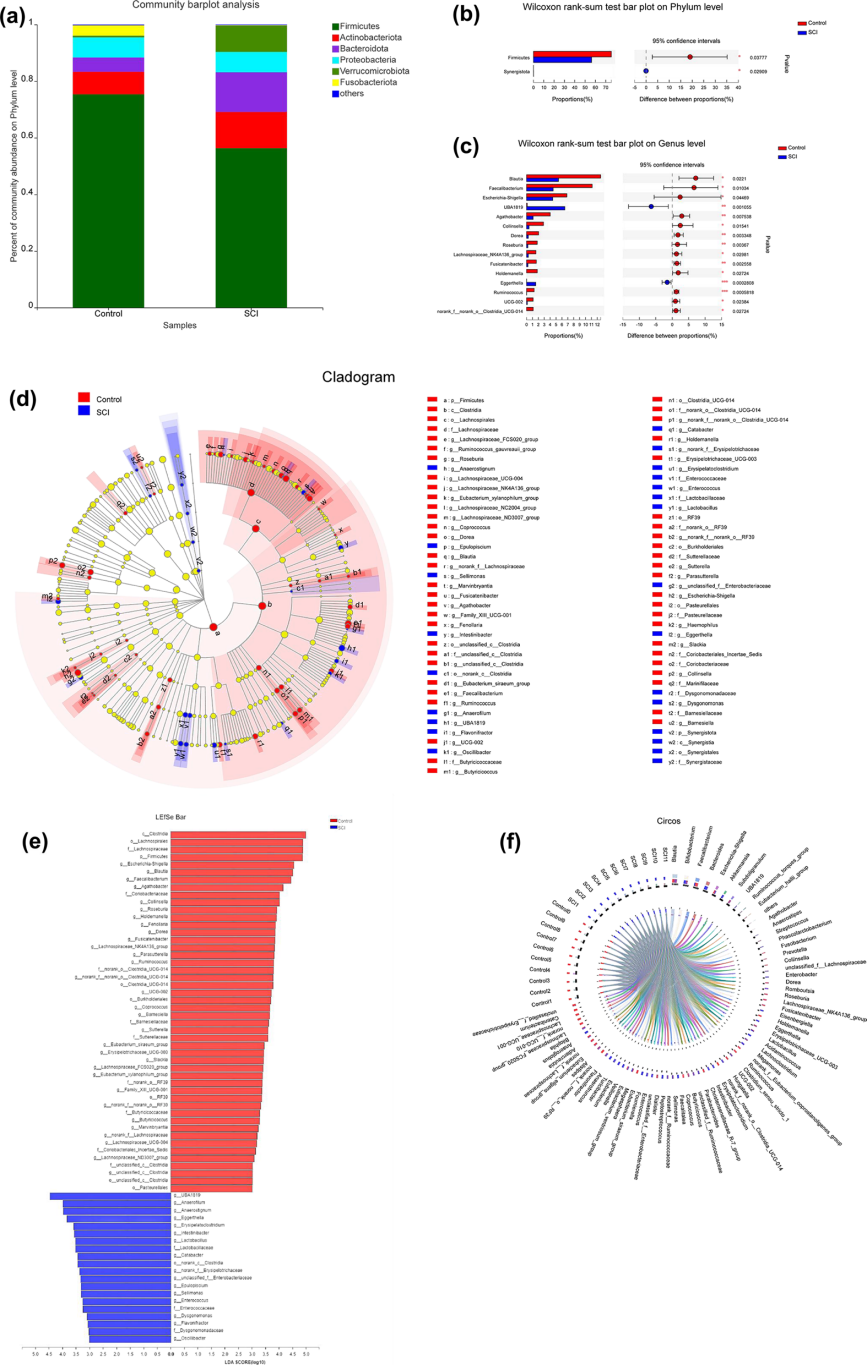
Alterations in the gut microbiota at the phylum and genus levels between the two groups. **a** The six most abundant species at the phylum level in the two groups. **b**, **c** Microbiota displaying significantly different abundances at the phylum and genus levels (*p < 0.05, **p < 0.01, ***p < 0.001). **d** A cladogram of linear discriminant analysis (LDA) effect size (LEfSe) results in the Control and spinal cord injury (SCI) groups. **e** Histogram of the LDA scores calculated for a differential abundance of functional profiles in the two groups. A LDA score cutoff of 3.0 was used to indicate a significant difference. Different colors represent different groups. **f** Correlation between fecal microbiota structure and samples

Further analysis was performed at different taxonomic levels based on the annotated species results. *Firmicutes*, *Actionbacteriota*, *Bacteroidetes*, and *Proteobacteria* were the most abundant phyla among the gut microbiota of both groups (Fig. 2a). In addition, compared with the healthy controls, the abundance of *Synergistota* was significantly increased in patients with SCI, whereas that of *Firmicutes* was significantly decreased (Fig. 2b). At the genus level, the abundance of *UBA1819* (LDA = 4.54) and *Eggerthella* (LDA = 3.88) was significantly increased in SCI patients relative to that in the healthy controls. The results also showed marked decreases in the abundances of *Blautia* (LDA = 4.51), *Faecalibacterium* (LDA = 4.57), *Escherichia*–*Shigella* (LDA = 4.41), *Agathobacter* (LDA = 4.13), *Collinsella* (LDA = 4.04), *Dorea* (LDA = 3.88), *Roseburia* (LDA = 3.97), *Lachnospiraceae*_ NK4A136 group (LDA = 3.82), *Fusicatenibacter* (LDA = 3.80), *Holdemanella* (LDA = 3.87), *Ruminococcus* (LDA = 3.81), UCG-002 (LDA = 3.74), and *Clostridia*_UCG-014 (LDA = 3.84) (Fig. 2c).

To further determine the specific gut microbiota components associated with SCI, LEfSe analysis was used to identify the gut microbiota components of both groups. The results revealed 68 components with different classification levels, 20 of which were enriched in SCI patients and 48 in the Control group (LDA > 3; *p* < 0.05, Fig. 2d, e). Classification results showed that the 20 species enriched in the SCI group belonged to the phyla *Firmicutes* (*n* = 16), *Proteobacteria* (*n* = 2), *Actinobacteriota* (*n* = 1), and *Bacteroidota* (*n* = 1), while the 48 species enriched in the Control group belonged to the phyla *Firmicutes* (*n* = 36), *Proteobacteria* (*n* = 6), *Actinobacteriota* (*n* = 4), and *Bacteroidota* (*n* = 2). The correlation between fecal microbiota structure and fecal samples is shown in Fig. 2f.

### The serum metabolite profile of both groups

**Fig. 3:**
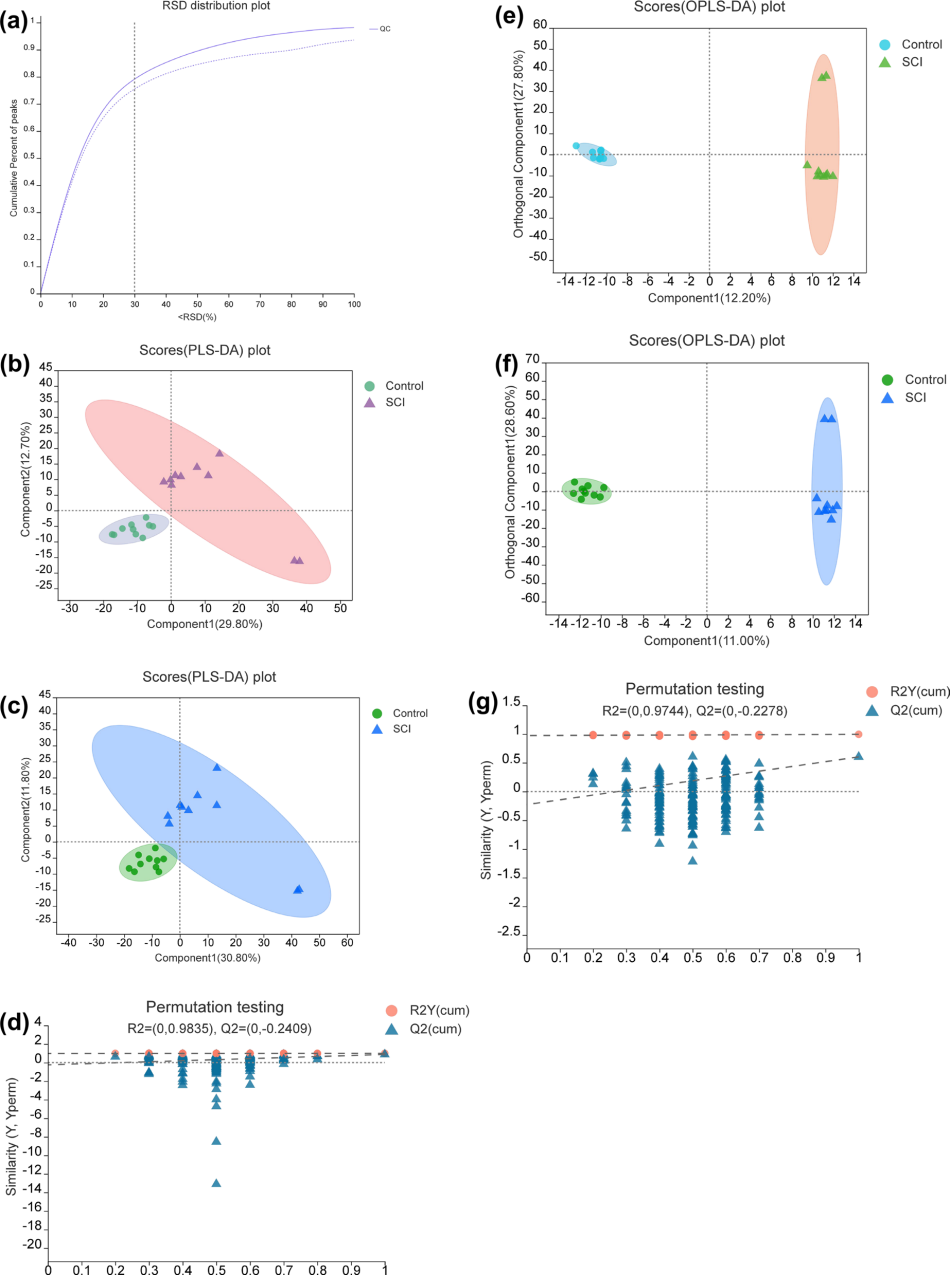
Changes in serum metabolite abundance in the Control and spinal cord injury (SCI) groups. **a** Relative standard deviation (RSD) distribution plot. **b**, **c** Partial least squares discriminant analysis (PLS-DA) score plots in positive ion mode and negative ion mode, respectively. **d** Model verification map of PLS-DA (permutation test). **e**, **f** Orthogonal PLS-DA (OPLS-DA) score plots in positive ion mode and negative ion mode, respectively. **g** Model verification map of OPLS-DA (permutation test)

To determine the extent of metabolic disorder resulting from SCI, untargeted metabolomics analysis was used to evaluate the differences in metabolite abundance between serum samples of the SCI group (*n* = 10) and those of the Control group (*n* = 10). In the metabolic profiles of all the samples, 5,039 positive and 4,894 negative model features were identified. As shown in Fig. 3a, when the relative standard deviation (RSD) was < 0.3, the peak proportion was > 70%, indicating that the sample size was appropriate. A comprehensive multivariate statistical analysis of cations and anions was undertaken using PLS-DA and OPLS-DA. In the PLS-DA (Fig. 3b, c) and OPLS-DA (Fig. 3e, f) score plots, a significant separation was observed between the Control and SCI groups, indicating that SCI led to metabolic dysfunction. Furthermore, permutation tests showed that the PLS-DA (Fig. 3d) and OPLS-DA (Fig. 3g) patterns had good reliability.

**Fig. 4:**
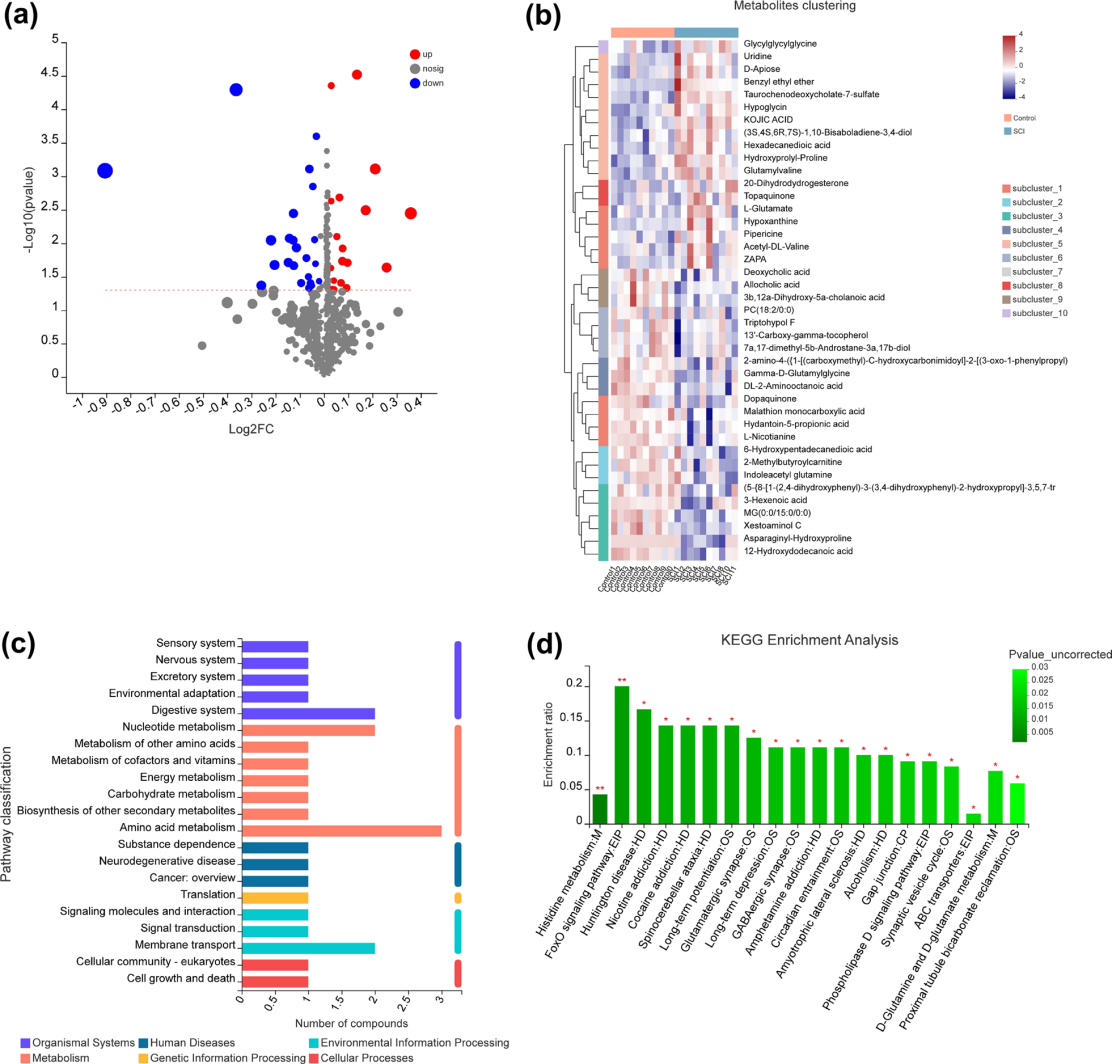
Differential metabolite extraction and KEGG pathway enrichment analysis. **a** Volcano plot of the differentially abundant metabolites. The abscissa is the multiple change value of the expression difference of metabolites between the two groups, and the ordinate is the statistical test value of the expression difference of metabolites (*p*-value). Each point in the figure represents a specific metabolite. **b** Heatmap of the differentially abundant metabolites between the two groups (variable importance in projection [VIP] scores > 3, *p* < 0.05). The color represents the relative abundance of the metabolites in the samples. **c** Level 1 and 2 KEGG pathways related to the differentially expressed metabolites. The ordinate is the name of the level 2 pathway and the abscissa is the number of metabolites related to that pathway. Different colors represent different level 1 pathways. **d** KEGG pathway enrichment column chart. The abscissa is the name of the level 3 pathway. *CP* cellular processes, *EIP* environmental information processing, *GIP* genetic information processing, *HD* human diseases, *M* metabolism, *OS* organismal systems

A total of 1511 differential metabolites (*p* < 0.05, VIP score > 1) were detected. Furthermore, 41 named differential metabolites were quantified. Forty-one metabolites exhibited significant differential abundance between the SCI patients and healthy controls, 18 of which were upregulated and 23 downregulated (Fig. 4a). The differentially abundant metabolites are listed in Table 2. The metabolites exhibiting significant differential abundance between the two groups are shown in the cluster heatmap in Fig. 4b.

**Table 2:** Differentially expressed serum metabolites between patients with SCI and healthy controls

Metabolite	Formula	Adducts	VIP	FC	*p*-value	Mode
PC(18:2/0:0)	C26H50NO7P	M + H-H2O, M + Na, M + H	1.05	0.99	0.037	pos
Benzyl ethyl ether	C9H12O	M + H, M + NH4, M + H-H2O	3.71	1.28	0.004	pos
Hypoxanthine	C5H4N4O	M + H-H2O, M + H	1.90	1.05	0.018	pos
3-Hexenoic acid	C6H10O2	M + ACN + H, 2 M + K	2.59	0.87	0.021	pos
*L*-Nicotianine	C10H12N2O4	M + H-H2O	2.79	0.86	0.009	pos
Indoleacetyl glutamine	C15H17N3O4	M + H	2.45	0.84	0.043	pos
Hypoglycin	C7H11NO2	M + H	1.12	1.02	0.002	pos
Pipericine	C22H41NO	M + H	1.04	1.02	0.023	pos
Xestoaminol C	C14H31NO	M + H	1.93	0.96	0.001	pos
12-Hydroxydodecanoic acid	C12H24O3	2M + NH4	1.40	0.98	0.000	pos
MG(0:0/15:0/0:0)	C18H36O4	M + NH4	4.37	0.78	0.000	pos
6-Hydroxypentadecanedioic acid	C15H28O5	M + ACN + H	1.99	0.92	0.022	pos
*DL*-2-Aminooctanoic acid	C8H17NO2	M + H	2.23	0.92	0.004	pos
2-Methylbutyroylcarnitine	C12H23NO4	M + H	1.30	0.97	0.009	pos
Glycylglycylglycine	C6H11N3O4	M + H-2H2O	1.57	1.05	0.012	pos
Hydroxyprolyl-proline	C10H16N2O4	M + H-H2O	2.60	1.13	0.003	pos
Dopaquinone	C9H9NO4	M + ACN + H	1.61	0.95	0.017	pos
KOJIC ACID	C6H6O4	M + H	1.28	1.02	0.000	pos
2-Amino-4-butanoic acid	C19H25N3O7S	M + 2Na-H	2.22	0.90	0.019	pos
ZAPA	C4H6N2O2S	M + H	1.54	1.05	0.039	pos
Oxidanesulfonic acid	C30H28O14S	M + 2Na-H	1.16	0.98	0.020	pos
13′-Carboxy-gamma-tocopherol	C28H46O4	M-H, M + Na-2H	1.51	0.96	0.039	neg
Glutamylvaline	C10H18N2O5	M-H2O-H	3.02	1.16	0.001	neg
Uridine	C9H12N2O6	M-H	1.16	1.03	0.049	neg
3b,12a-Dihydroxy-5a-cholanoic acid	C24H40O4	M + FA-H	2.17	0.91	0.008	neg
Deoxycholic acid	C24H40O4	M-H	1.70	0.94	0.039	neg
Topaquinone	C9H9NO5	M-H2O-H	1.54	1.07	0.046	neg
Allocholic acid	C24H40O5	M-H	2.20	0.92	0.012	neg
1,10-Bisaboladiene-3,4-diol	C15H26O2	M + FA-H	1.34	1.04	0.008	neg
Triptohypol F	C31H52O2	M + FA-H	1.39	0.96	0.043	neg
Malathion monocarboxylic acid	C8H15O6PS2	M + FA-H	1.35	0.96	0.032	neg
20-Dihydrodydrogesterone	C23H34	M + Na-2H	2.51	1.20	0.023	neg
7a,17-dimethyl-5b-Androstane-	C21H36O2	M + FA-H	1.45	0.96	0.046	neg
3a,17b-diol						
Taurochenodeoxycholate-7-sulfate	C26H45NO9S2	M-2H	2.54	1.10	0.000	neg
Acetyl-*DL*-Valine	C7H13NO3	M-H	1.08	1.03	0.036	neg
Hydantoin-5-propionic acid	C6H8N2O4	M-H	2.00	0.92	0.009	neg
Asparaginyl-hydroxyproline	C9H15N3O5	M + FA-H	6.25	0.53	0.001	neg
Gamma-*D*-glutamylglycine	C7H12N2O5	M-H	1.47	0.97	0.001	neg
*D*-Apiose	C5H10O5	M-H	1.75	1.07	0.020	neg
Hexadecanedioic acid	C16H30O4	M-H	1.61	1.05	0.002	neg
*L*-Glutamate	C5H9NO4	M-H	1.04	1.02	0.048	neg

### Differential metabolites and KEGG pathway enrichment analysis

We next applied KEGG pathway enrichment analysis to the 41 named differential metabolites. The results suggested that the altered metabolites were mainly related to amino acid metabolism, digestive system, nucleotide metabolism, and membrane transport (Fig. 4c). The 41 metabolites were enriched in 20 KEGG pathways (*p* < 0.05). Histidine metabolism: M and FoxO signaling pathway: EIP were the two most significantly enriched pathways (*p* < 0.01, Fig. 4d).

### Analysis of the correlations among gut dysbiosis, altered serum metabolites, and clinical parameters

**Fig. 5:**
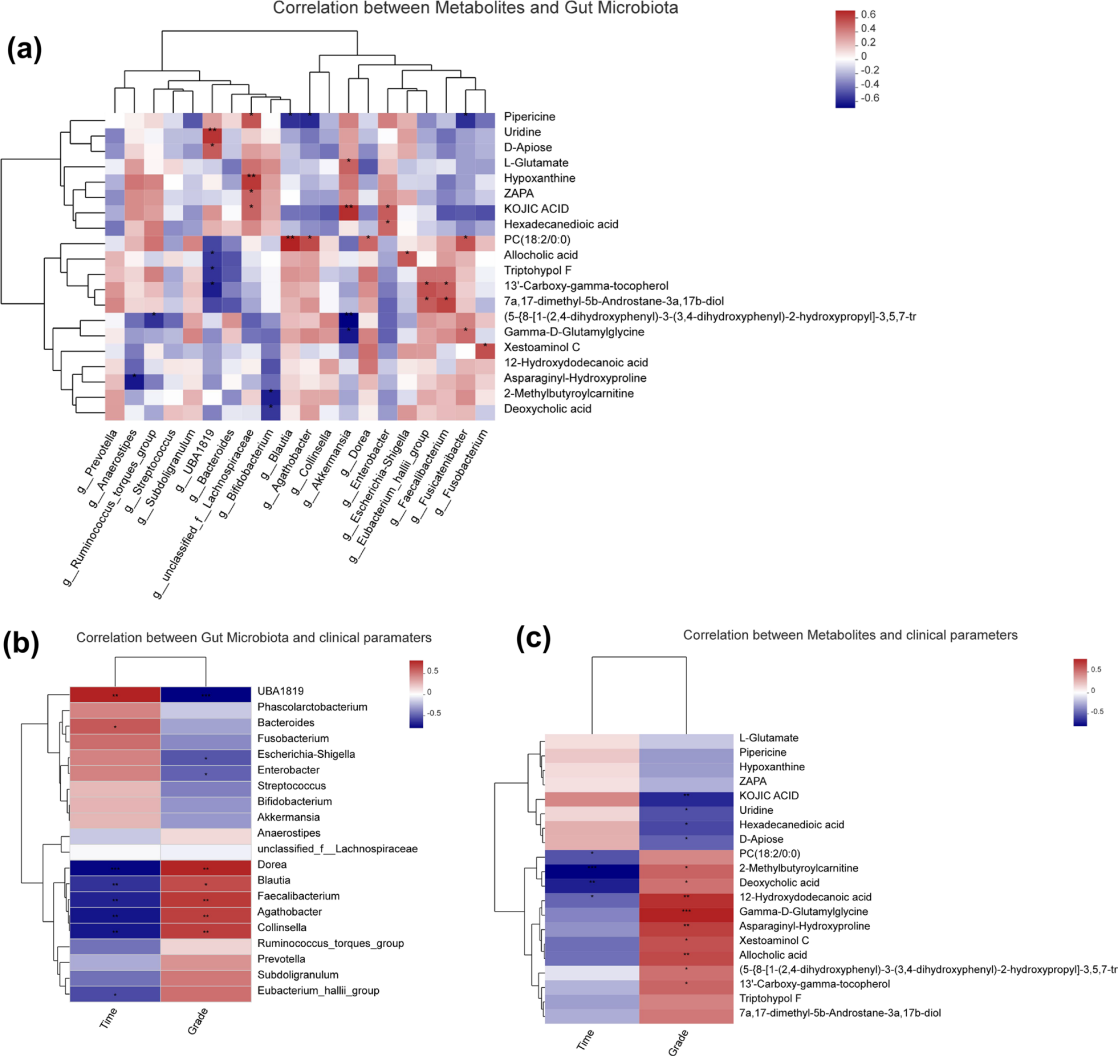
Analysis of the correlation among the gut microbiota, serum metabolites, and clinical parameters. **a** Heatmap of the correlations between differentially abundant species and metabolites (associations between 41 differentially abundant serum metabolites and the 20 most abundant genera). **b** Heatmap of the correlations between the gut microbiome and clinical parameters. **c** Heatmap of the correlations between metabolites and clinical parameters

Spearman’s correlation was used to investigate the relationship between gut microbiota and metabolites. The relationship between the 20 most differentially expressed metabolites and the 20 most differentially abundant gut microbiota at the genus level was analyzed in patients with SCI (Fig. 5a). Significant correlations were found between *UBA1819* and uridine (*C* = 0.609, *p* = 0.004), *Lachnospiraceae* and hypoxanthine (*C* = 0.595, *p* = 0.006), *Blautia* and PC (18:2/0:0) (*C* = 0.659, *p* = 0.002), and *Akkermansia* and kojic acid (*C* = 0.628, *p* = 0.003). Meanwhile, the abundance of *Akkermansia* was significantly and negatively correlated with that of (5-{8-[1-(2,4-dihydroxyphenyl)-3-(3,4-dihydroxyphenyl)-2-hydroxypropyl]-3,5,7-trih-ydroxy-3,4-dihydro-2H-1-benzopyran-2-yl}-2-hydroxyphenyl) oxidanesulfonic acid in serum (*C* = 0.609, *p* = 0.004).

To explore the clinical significance of gut microbiome and metabolite dysregulation in patients with SCI, the putative correlations among gut microbiota abundance, metabolite abundance, and clinical parameters (including injury duration and neurological grade) were analyzed using Spearman’s correlation (Fig. 5b, c). We found that neurological grade was significantly and positively correlated with the abundance of *Dorea* (*C* = 0.655, *p* = 0.001), *Faecalibacterium* (*C* = 0.587, *p* = 0.005), *Agathobacter* (*C* = 0.567, *p* = 0.007), and *Collinsella* (*C* = 0.575, *p* = 0.006) and significantly and negatively correlated with the abundance of *UBA1819* (*C* =  − 0.709, *p* = 0.000). Simultaneously, neurological grade was identified as being significantly and positively correlated with the level of 12-hydroxydodecanoic acid (*C* = 0.455, *p* = 0.001), gamma-*D*-glutamylglycine (*C* = 0.712, *p* = 0.000), asparaginyl-hydroxyproline (*C* = 0.613, *p* = 0.004), and allocholic acid (*C* = 0.581, *p* = 0.007) and significantly and negatively correlated with the level of kojic acid (*C* =  − 0.617, *p* = 0.004). In addition, injury duration showed a significant positive correlation with the abundance of *UBA1819* (*C* = 0.660, *p* = 0.001) and a significant negative correlation with the abundance of *Dorea* (*C* =  − 0.691, *p* = 0.001), *Blautia* (*C* = 0.575, *p* = 0.006), *Faecalibacterium* (*C* =  − 0.618, *p* = 0.003), *Agathobacter* (*C* = 0.652, *p* = 0.001), and *Collinsella* (*C* = 0.646, *p* = 0.002). Meanwhile, injury duration was significantly and negatively correlated with the level of 2-methylbutyroylcarnitine (*C* =  − 0.730, *p* = 0.000) and deoxycholic acid (*C* = 0.642, *p* = 0.002). The overall correlation results suggested that gut microorganisms *UBA1819*, *Dorea*, *Faecalibacterium, Agathobacter,* and *Collinsella* and the metabolites kojic acid, 12-hydroxydodecanoic acid, gamma-*D*-glutamylglycine, asparaginyl-hydroxyproline, and allocholic acid were associated with neurological grade. The results further suggested that the gut microbes *UBA1819*, *Dorea*, *Blautia*, *Faecalibacterium*, *Agathobacter*, and *Collinsella* and the metabolites 2-methylbutyroylcarnitine and deoxycholic acid were related to injury duration.

## Discussion

In this study, we compared the gut microbiota and serum metabolites of patients with SCI and healthy individuals using 16S rRNA sequencing and metabolomics. The patients and the controls recruited for our study were age- and gender-matched and were from the same geographical location. Despite these similarities, we found that gut microbiota and metabolite composition was significantly altered in SCI patients compared with that in the controls, and the changes were correlated. The main findings of our study were as follows: (1) The composition of the gut microbiota differed between SCI patients and healthy individuals, suggesting that SCI induced gut dysbiosis; (2) the serum metabolite profile of patients with SCI differed from that of healthy individuals, implying that SCI patients experience metabolic abnormalities; (3) the gut microbiota dysregulation that occurs following SCI is closely related to the changes in the serum metabolite profile as well as clinical parameters (including injury duration and neurological grade). The metabolomics analysis identified many metabolites that are closely associated with CNS diseases and may be important therapeutic targets for the treatment of SCI, a notion that merits further investigation.

Increasing evidence, both basic and clinical, has indicated that the gut microbiome is involved in regulating a variety of cellular and molecular processes under both physiological and pathological conditions. The gut microbiome and its metabolites can migrate from the gut to the intestinal wall and cross the intestinal barrier, thereby promoting inflammation and affecting other organs [24]. However, the precise mechanisms underlying these effects are unclear. Immune-, endocrine-, metabolism-, and neurotransmitter-related pathways are considered to be the main mechanisms via which the gut microbiota influence the occurrence and development of CNS diseases. In the last decade, an increasing number of studies have focused on the role of gut microbes in regulating CNS function, especially in animal models. One study showed that dysregulation of the gut microbiome is associated with impaired functional recovery and increased anxiety-like behavior after SCI [25]. Meanwhile, Jing et al. found that fecal microbiota transplantation can help restore the gut microbiota and the metabolite profile of mice with SCI, thereby improving intestinal and neurological function [26]. A different study reported that the feeding of commercial probiotics to mice promoted anti-inflammatory responses by increasing the number of regulatory T cells, thus alleviating neuropathology and promoting motor recovery in the animals [18]. The same study also demonstrated that lactic acid supplementation facilitates functional recovery after SCI. These results suggested that the gut microbiome is a key regulator of SCI pathogenesis. The modulation of the gut microbiome and derived metabolites in SCI patients is expected to reduce functional impairment and promote nerve regeneration.

At the phylum level, the gut microbiome primarily consists of *Firmicutes*, *Bacteroidetes*, *Actinobacteria*, *Proteobacteria*, and *Verrucomicrobia* [27]. *Firmicutes* and *Bacteroidetes* were reported to account for approximately 90% of all bacteria in the gut [28]. These bacteria ferment indigestible polysaccharides and produce metabolites that can be used as energy by the host. The relative abundance of some constituents of the gut microbiome was reported to be altered in patients with SCI [29]. In a Chinese cohort study involving patients with chronic traumatic complete SCI, Zhang et al. observed that the proportions of *Proteobacteria* and *Verrucomicrobia* were increased, while that of *Bacteroidetes* was decreased [6]. Another clinical study that enrolled 54 Turkish people (41 patients with SCI and 13 healthy individuals) reported that SCI patients had a significantly reduced abundance of *Firmicutes* compared with healthy individuals [17]. However, results from animal experiments have indicated that the abundance of *Firmicutes* is decreased and that of *Bacteroidetes* increased in mice with SCI and that *Firmicutes* is positively correlated with motor recovery [14, 30]. Similarly, the number of *Bacteroidetes* was reported to be increased in mice with acute or subacute SCI [18, 31]. Consistent with these studies, we also found significant changes in the composition of the gut microbiome in patients with SCI, namely, the abundance of *Firmicutes* was found to be decreased, whereas that of *Synergistota* was increased, in SCI patients. Our results related to the changes in gut microbiota composition after SCI are not entirely consistent with those of previous studies. Possible reasons for this include (1) differences in the severity of SCI, such as between complete and incomplete injury [32]; (2) differences in diet, environment, and gut microbe composition among individuals; (3) differences in antibiotic intake; and (4) experimental bias. To reduce the influence of confounding factors on our results, the two groups of participants enrolled in our study were matched for gender and age and all originated from the same region. Additionally, dietary management was standardized before sampling.

The gut microbiota regulates several key metabolic processes in the body; accordingly, gut microbiota imbalance is a major contributor to metabolic disorders in the host [27]. Clinical studies and studies involving animals have demonstrated that changes in the gut microbiome after SCI are closely linked to metabolic abnormalities. For instance, changes in the gut microbiome of patients with thoracic SCI were reported to be correlated with differences in serum biomarker levels, suggesting that gut dysbiosis was associated with multiple metabolic processes [32]. It has also been suggested that changes in the abundance of *Bacteroidetes* and *Firmicutes* after SCI may cause or lead to chronic metabolic disorder [32, 33]. Moreover, studies have shown that people with paraplegia or quadriplegia have significantly impaired metabolic function and higher body fat content than healthy people [33, 34]. Similar to the results of previous studies, our untargeted metabolomics analysis indicated that the serum metabolite profile was significantly altered in patients with SCI relative to that in the healthy controls and that this was correlated with changes in the gut microbiome.

In our study, 68 differentially abundant microbial strains and 41 differentially expressed metabolites were selected for association analysis and a combined analysis with clinical indicators. The abundance of *UBA1819*, *Lachnospiraceae*, *Blautia*, and *Akkermansia* was found to be significantly and positively correlated with that of serum uridine, kojic acid, hypoxanthine, and PC(18:2/0:0), respectively. Additionally, the increase in *UBA1819* abundance observed in patients with SCI was positively correlated with injury duration, but negatively correlated with motor function. Importantly, the abundance of UAB1819 was positively correlated with serum uridine levels. *UBA1819* belongs to the family *Ruminococcaceae* [35], the members of which produce SCFAs that help maintain the health of the digestive tract [36]. It has been shown that *Ruminococcaceae* is enriched in behavior-deficient mice and is closely associated with anxiety-like behavior [37]. In our study, we found that *UBA1819* abundance was increased after SCI, which may be associated with chronic motor dysfunction and depression. Uridine is a precursor of the pyrimidine nucleotides necessary for RNA synthesis as well as an essential starting material for many metabolic processes. Uridine has many critical biological functions. For instance, it has been reported that uridine can reduce inflammation and oxidative stress levels [38], reduce cytotoxicity [39], and improve neurophysiological functions [40, 41]. Uridine can also influence regeneration in a variety of mammalian tissues and promote stem cell activity [42]. These observations imply that *UBA1819* may play a role in SCI by influencing uridine metabolism and that countering oxidative stress and promoting nerve regeneration may serve to ameliorate the pathology of SCI.

In addition to uridine, hypoxanthine and kojic acid, identified as being differentially abundant in this study, are also closely related to neurological diseases. Hypoxanthine increases the expression of proinflammatory cytokines and that of NF-κB, decreases nitrite levels, and induces microglia and astrocyte activation [43]. Hypoxanthine and kojic acid have both been associated with the induction of oxidative stress. Moreover, kojic acid was reported to exert a significant inhibitory effect on glial cell activation and the release of inflammatory factors [44, 45]. These metabolites may serve as important therapeutic targets for SCI, a possibility that will be examined in subsequent studies.

Despite the importance of our findings, this study nevertheless had several limitations. The first was that it is not possible to eliminate the influence of confounding factors given that the factors that can affect the composition of the gut microbiota and metabolite abundance are extremely complex. The second limitation of this study was that, although the results indicated that the sampling of the microbial community was adequate, the sample size was still small. The third limitation was the lack of verification of the differential flora and metabolite abundance. However, this study was the first to use serum metabolomics combined with the gut microbiota to study the relationship between the intestinal environment and clinical parameters in patients with SCI. Importantly, we found that uridine, glutamate, hypoxanthine, and kojic acid are closely related to SCI and represent potentially important targets for the treatment of this condition. Our data provided a novel perspective for the study of the gut microbiota and associated metabolism after SCI.

## Data Availability

The data that support the findings in this study are available from the corresponding author upon reasonable request.

## Abbreviations

SCI: Spinal cord injury
CNS: Central nervous system
SCFAs: Short-chain fatty acids
ASIA: American Spinal Cord Injury Association
CTAB: Cetyltrimethyl ammonium bromide
OTUs: Operational taxonomic units
RDP: Ribosomal Database Project
PCA: Principal component analysis
PCoA: Principal coordinate analysis
PLS-DA: Partial least squares discriminant analysis
QIIME: Quantitative insights into microbial ecology
LDA: Linear discriminant analysis
ANOSIM: Analysis of similarity
ESI: Electrospray ionization
OPLS-DA: Orthogonal PLS-DA
FC: Fold change
